# Gender differences in microRNA expression in levodopa-naive PD patients

**DOI:** 10.1007/s00415-023-11707-0

**Published:** 2023-04-13

**Authors:** A. Vallelunga, T. Iannitti, G. Somma, M. C. Russillo, M. Picillo, R. De Micco, L. Vacca, R. Cilia, C. E. Cicero, R. Zangaglia, G. Lazzeri, S. Galantucci, F. G. Radicati, A. De Rosa, M. Amboni, C. Scaglione, A. Tessitore, F. Stocchi, R. Eleopra, A. Nicoletti, C. Pacchetti, A. Di Fonzo, M. A. Volontè, P. Barone, M. T. Pellecchia

**Affiliations:** 1 Department of Life Sciences and Biotechnologies, Section of Medicines and Health Products, University of Ferrara, Ferrara, Italy; 2 Department of Medical Sciences, Section of Experimental Medicine, University of Ferrara, Ferrara, Italy; 3 Department of Medicine Surgery and Dentistry “Scuola Medica Salernitana”, Neuroscience Section, University of Salerno, Fisciano, Italy; 4 Department of Advanced Medical and Surgical Sciences, University of Campania “Luigi Vanvitelli”, Naples, Italy; 5 IRCCS San Raffaele, Rome, Italy; 6 Department of Clinical Neurosciences, Parkinson and Movement Disorders Unit, Fondazione IRCCS Istituto Neurologico Carlo Besta, Milan, Italy; 7 Neurologic Unit, AOU “Policlinico-San Marco”, Department of Medical, Surgical Sciences and Advanced Technologies, GF Ingrassia, University of Catania, Catania, Italy; 8 Parkinson’s Disease and Movement Disorders Unit, IRCCS Mondino Foundation, Pavia, Italy; 9 IRCCS Ca’ Granda Ospedale Maggiore Policlinico, Neurology Unit, Milan, Italy; 10 IRCCS San Raffaele Scientific Institute, Neurology Unit, Milan, Italy; 11 Department of Neurosciences and Reproductive and Odontostomatological Sciences, Federico II University, Naples, Italy; 12 IRCCS Istituto Delle Scienze Neurologiche di Bologna, Bologna, Italy; 13 University San Raffaele, Roma, Italy

**Keywords:** Parkinson’s disease, miRNAs, Gender differences, Target genes

## Abstract

Gender is an important factor influencing epidemiological and clinical features of Parkinson’s disease (PD). We aimed to evaluate gender differences in the expression of a panel of miRNAs (miR-34a-5p, miR-146a, miR-155, miR-29a, miR-106a) possibly involved in the pathophysiology or progression of disease. Serum samples were obtained from 104 PD patients (58 men and 46 women) never treated with levodopa. We measured levels of miRNAs using quantitative PCR. Correlations between miRNA expression and clinical data were assessed using the Spearman’s correlation test. We used STRING to evaluate co-expression relationship among target genes. MiR-34a-5p was significantly upregulated in PD male patients compared to PD female patients (fc: 1.62; *p* < 0.0001). No correlation was found with age, BMI, and disease severity, assessed by UPDRS III scale, in male and female patients. MiR-146a-5p was significantly upregulated in female as compared to male patients (fc: 3.44; *p* < 0.0001) and a significant correlation was also observed between disease duration and mir-146a-5p. No differences were found in the expression of miR-29a, miR-106a-5p and miR-155 between genders. Predicted target genes for miR-34a-5p and miR-146-5p and protein interactions in biological processes were reported. Our study supports the hypothesis that there are gender-specific differences in serum miRNAs expression in PD patients. Follow-up of this cohort is needed to understand if these differences may affect disease progression and response to treatment.

**Supplementary Information:**

The online version contains supplementary material available at 10.1007/s00415-023-11707-0.

## Introduction

Parkinson’s disease (PD) is the second most common neurodegenerative disorder and affects millions of individuals worldwide. Gender differences have been recognized in PD, both in epidemiological data, with men being 1.5 more likely to develop the disease compared to women, and in the incidence of motor and non-motor symptoms and the disease progression [1–4]. Since it is now widely known that PD is not a single disease, detection of biomarkers that may characterize patient groups and predict disease progression and outcome for the purpose of personalized medicine is the goal to strive for. A personalized therapeutic approach also considering gender differences would be advisable to improve PD patients’ quality of life [5].

MicroRNAs (miRNAs) are small non-coding RNAs which regulate gene expression at post-transcriptional level. MiRNAs can cross the blood–brain barrier and enter body fluids as blood, urine and saliva. In addition, miRNAs are stable, easily quantifiable and accessible by minimally-invasive procedures [6] Several studies have shown that specific panels of miRNAs are dysregulated in PD and other parkinsonian disorders [7, 8]. Such evidence shows that the use of miRNAs as biomarkers has great potential for early PD diagnosis.

Generally, deregulated miRNAs show consistent expression between men and women, but some miRNAs may be differentially expressed between genders in specific diseases [9]. These differentially expressed miRNAs have important roles in basic biological processes and contribute to the development and progression of diseases. So far, no gender-oriented analysis of miRNA panels has been performed in early PD.

Our aim was to evaluate gender differences in the expression of some miRNAs that, according to the literature, are possibly involved in the pathophysiology and progression of PD.

## Materials and methods

### Patients and sample collection

PD patients never treated with levodopa were recruited from 11 Italian centers. We enrolled 105 patients affected by PD (59 men and 45 women). Clinical features were reported in Table 1. The study protocol was approved by Ethics Committees at all participant centers (University of Salerno, Salerno; University of Campania “Luigi Vanvitelli”, Napoli; IRCCS San Raffaele, Roma; Fondazione IRCCS Istituto Neurologico Carlo Besta, Milan; AOU “Policlinico-San Marco”, Catania; IRCCS Mondino Foundation, Pavia; IRCCS Ca' Granda Ospedale Maggiore Policlinico, Milan; IRCCS San Raffaele Scientific Institute, Milan; University Federico II, Naples; IRCCS Istituto delle Scienze Neurologiche, Bologna; University San Raffaele, Roma). Written informed consent was obtained from all patients. Blood samples were obtained by vein puncture using dry vacutainer tubes (BD Biosciences, Italy). Each sample was processed for serum isolation within 2 h after withdrawal according to our previously published protocol [10, 11].

**Table 1:** Demographic and clinical features of study population at baseline

	Men (*n* = 59)	Women (*n* = 45)	*p*
Age, ys (mean ± SD)	64.52 (9.32)	64.43 (9.61)	NS
Disease duration, months (mean ± SD)	26.24 (21.90)	28.41 (25.52)	NS
MDS-UPDRS III (mean ± SD)	27.37 (9.46)	28.53 (14.39)	NS
H&Y stage	1.90 (0.48)	1.93 (0.65)	NS

### MIRNAS quantification

We assessed hemolysis grade according to Shah et al., which quantified the ratio of miR-451a and miR-23a-3p to determine the samples with low (miR ratio < 5), moderate (5 < miR ratio > 7) and severe (miR > 7) grade of hemolysis [12]. Then we excluded all samples with severe hemolysis. Serum miR-34a-5p and miR-146a-5p were quantified using LNATM enhanced microRNA assay (Exiqon) according to our previous published protocol [10, 11]. Each miRNA was quantified in duplicate and mean Ct values were used for fold change calculations.

### Statistical analysis

We used miR-93-5p as reference miRNA according to our previously published protocol [8]. The data was checked for normality using the Anderson Darling test and analyzed using parametric or nonparametric tests accordingly. Correlations between miRNA expression and clinical features were assessed using the Spearman’s correlation test. We excluded samples with Ct values higher than 37 from the analysis. We calculated the fold changes (fc) using the 2 − 1CT method for miR-34a-5p and miR-146a-5p. All statistical analyses were performed using GraphPad Prism (GraphPad Software Inc., San Diego, CA, USA). A p < 0.05 was considered significant.

### Target prediction

Target prediction of miRNAs that were found to be differentially expressed according to gender was obtained by querying the microRNA-target interactions using the miRTarBase, chosen due to its widespread use and completeness. We considered only strong mRNA-miRNA interactions experimentally confirmed by qRT-PCR, luciferase assays and Western Blots. Then, we used the search tool for retrieval of interacting genes (STRING) to evaluate co-expression relationships among target genes. We considered only the target genes with co-expression coefficients > 0.7. Furthermore, we used open target platform (https://platform.opentargets.org/) to evaluate if predicted targets were associated to PD.

## Results

### Serum MIR-34a-5p

**Fig. 1:**
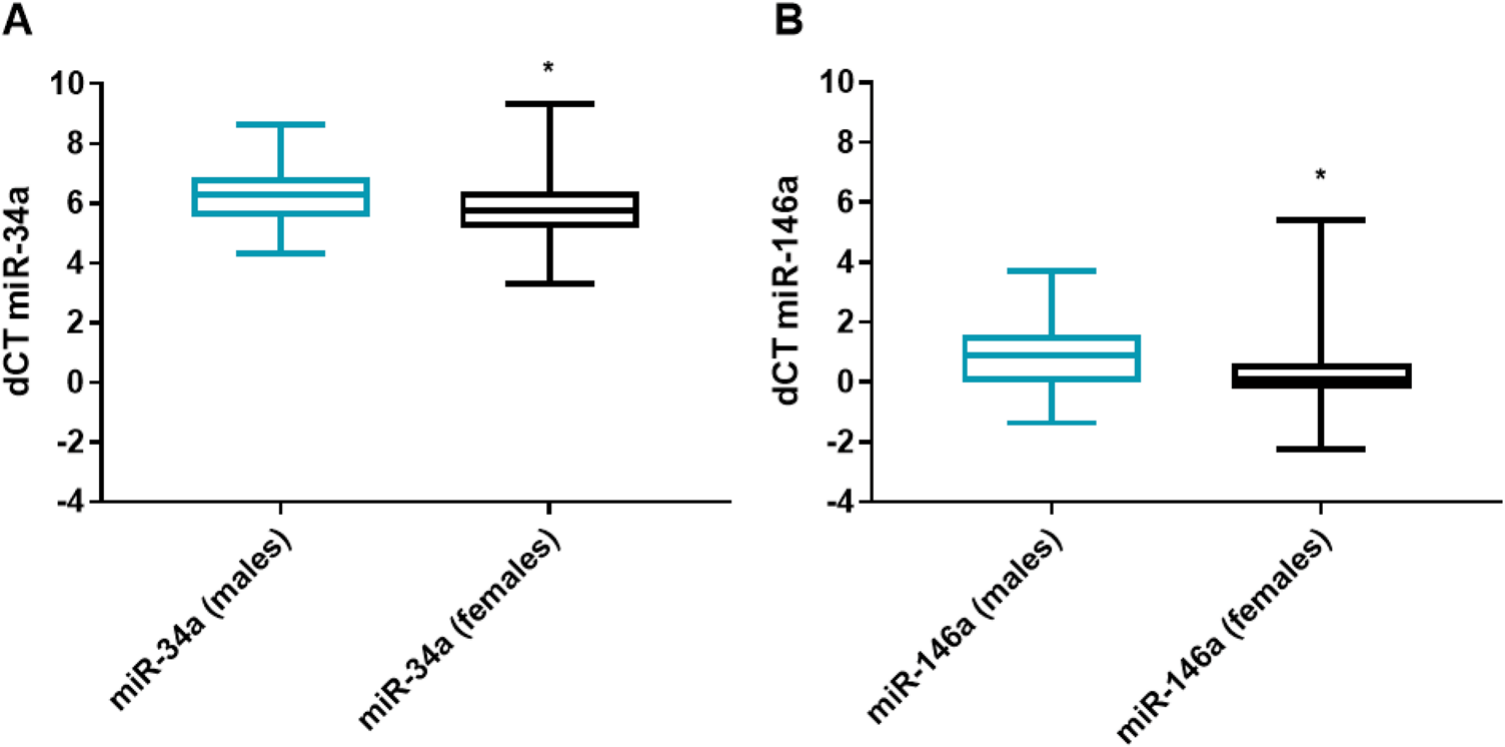
Box plots showing significant upregulation of miR-34a-5p (**A**) and miR-146a-5p (**B**) in PD male patients compared with PD female patients (fc: 1.62; *p* < 0.0001; fc: 3,44; *p* < 0.0001, respectively)

**Fig. 2:**
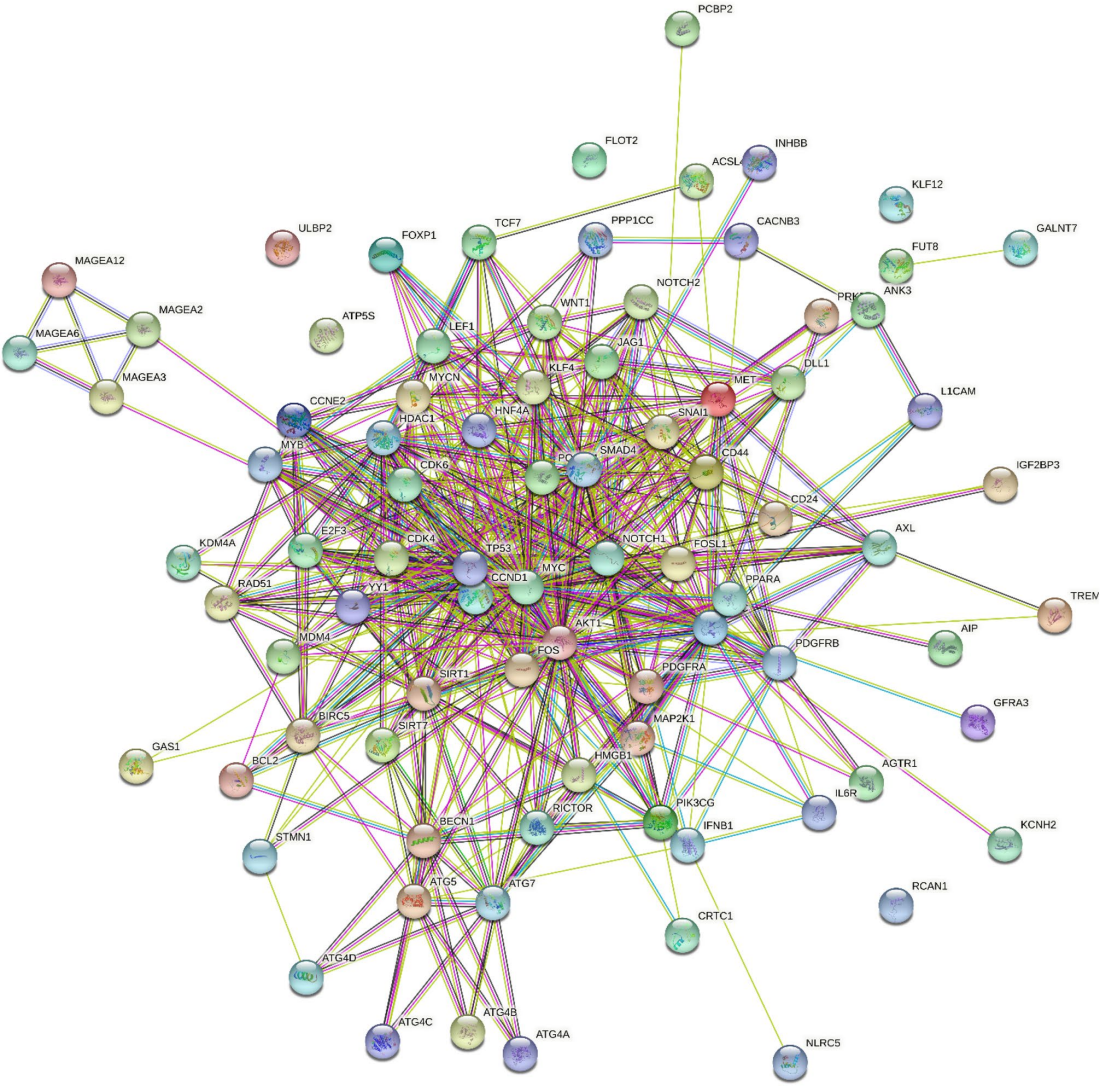
Co-expression relationships among target genes of miR-34a-5p derived from the search tool for retrieval of interacting genes (STRING)

**Fig. 3:**
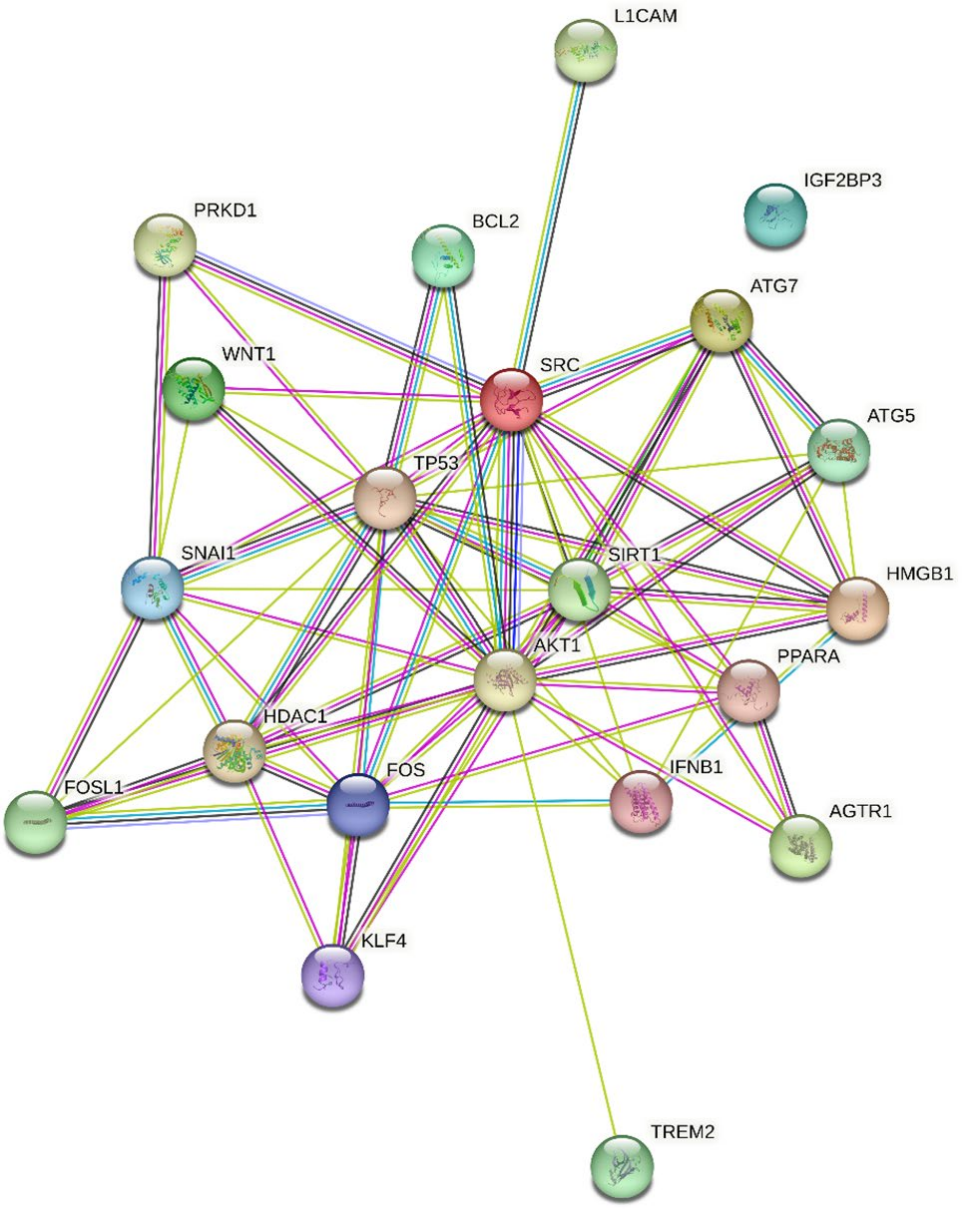
Target genes of miR-34a-5p associated to PD obtained using open target platform

We found that miR-34a-5p was significantly upregulated in PD men patients compared with PD women patients (fc: 1.62; p < 0.0001) (Fig. 1A). We did not find any correlation with age, BMI, and disease severity, assessed by UPDRS III scale, in men and women. Using miRTarBase, we identified 85 target genes, confirming strong mRNA-miRNA interactions for miR-34a-5p. Many predicted target genes are involved in neurodegeneration. Using STRING, we found several interactions among target proteins (Fig. 2). We observed that 9 target genes are involved in aging [False Discovery Rate (FDR) = 0.00011]. In addition, 15 target genes are implicated in the regulation of neurogenesis (FDR = 0.000008) and 10 genes are involved in the regulation of neuronal death (FDR = 0.00084) (Table 2) Using open target platform, we identified 22 target genes associated to PD (Fig. 3).

**Table 2:** Target genes of miR-34a-5p involved in neurodegeneration

Biological process	Target genes	False discovery rate
Aging	MAGEA2, TP53, AKT1, BCL2, SIRT1, FOS, AKT1, PDGFRA, PDGFR, MAP2K1, ATG7	0.00011
Regulation of neurogenesis	PP1CC, PRKD1, L1CAM, DLL1, JAG1, KLF4, HDAC1, MYB, TP53, NOTCH1, TREM, AKT1, BCL2, MAP2K1, CRTC1	0.000008
Regulation of neuronal death	WNT1, MYB, TP53, AXL, PPARA, FOS, SIRT1, BCL2, ATG7, AKT1	0.00084

### Serum MIR-146a-5p

We observed that miR-146a-5p was significantly upregulated in PD men compared with PD women (fc: 3.44; *p* < 0.0001) (Fig. 1B). A weak but significant correlation was observed between disease duration and mir-146a-5p (*r* = 0.2744; p < 0.05) only in male patients. No correlation.was found between miR-146a-5p and age, BMI in PD patients of both genders.

**Fig. 4:**
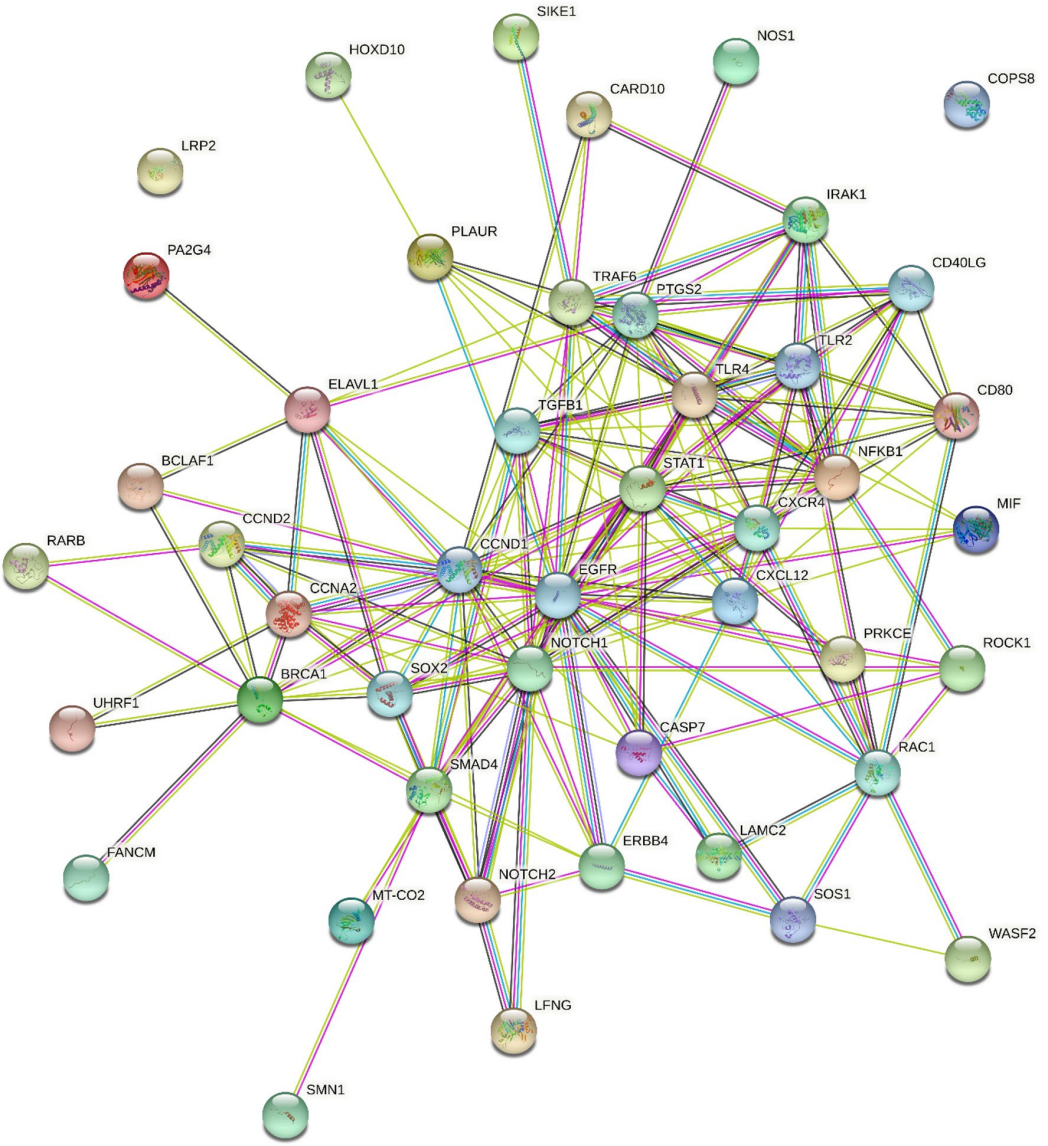
Co-expression relationships among target genes of miR-146a-5p derived from the search tool for retrieval of interacting genes (STRING)

**Fig. 5:**
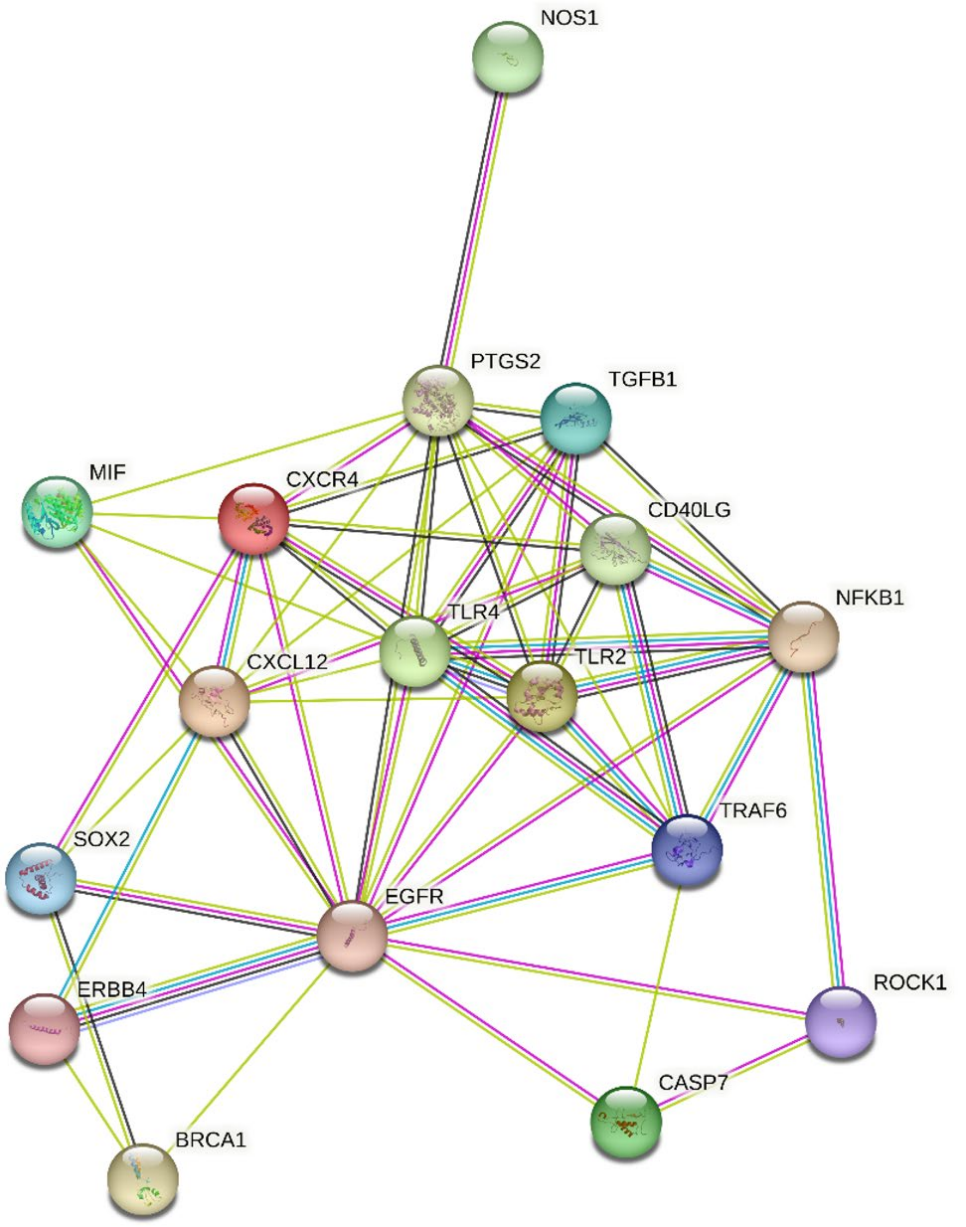
Target genes of miR-146a-5p associated to PD obtained using open target platform

Using miRTarBase, we identified 47 target genes confirming strong mRNA-miRNA interactions for miR-146a-5p. Many predicted target genes are involved in neurodegenerative processes. Using STRING, we found several interactions among target proteins (Fig. 4). We observed that 19 target genes are involved in neurogenesis (FDR = 0.000007) and 11 genes in the regulation of neurogenesis (FDR = 0.00013). In addition, 8 target genes are implicated in the process of axon guidance (FDR = 0.0000017) (Table 3). Using open target platform, we identified 17 target genes possibly associated to PD (Fig. 5).

**Table 3:** Target genes of miR-146a-5p involved in neurodegeneration

Biological process	Target genes	False discovery rate
Axon guidance	SMAD4, NOTCH2, SOS1, RAC1, LAMC2, CXCL12, CXCR4, NOTCH1	0.0000017
Neurogenesis	LRP2, HOXD10, NOS1, RARB, TGFB1, TLR2, TLR4, CXCR4, EGFR, CXCL12, SOX2, NOTCH1, ROCK1, SMAD4, NOTCH2, ERBB4, LAMC2, RAC1, SOS1	0.000007
Regulation of neurogenesis	LRP2, NOS1, TGFB1, TLR2, RARB, CXCR4, SOX2, NOTCH1, CXCL12, ROCK1, ERBB4	0.00013

No differences were found in the expression of miR-29a, miR-106a-5p and miR-155 between genders.

## Discussion

In this study, we evaluated gender differences in the expression of some miRNAs that, are possibly involved in the pathophysiology and progression of PD. MiR-34a-5p is abundantly expressed in the adult mammalian brain and we selected this miRNA because of its involvement in several neurodegenerative disorders, like Alzheimer's disease (AD), schizophrenia and major depression [13]. Specific deregulation of miR-34a-5p was found in cellular and animal models of PD but also in the blood and brain of PD patients [14, 15]. Recently, Grossi et al. found that miR-34a-5p was upregulated in plasmatic pure extracellular vesicles of PD patients. Furthermore, they observed that the levels of miR-34a-5p were associated with disease duration, Hoehn and Yahr and Beck Depression Inventory scores [16]. Mir-146a has a key role in inflammatory responses and is expressed within neurons, astrocytes and microglia [17]. Several reports have shown a downregulation of miR-146a-5p in PD patient-derived samples. Caggiu et al. found that miR-146a was downregulated in PD patients under levodopa treatment compared with healthy controls (HC) [18]. In another study, a group of 20 sporadic PD patients and 45 PD patients with mutations in the LRRK2 gene also found a decrease in levels of circulating miR146a in such patients [19]. MiR-155 is considered a pro-inflammatory mediator in the CNS and has a central role in the inflammatory response to α-syn in the brain [20]. Caggiu et al. reported that miRNA-155-5p was generally up-regulated in PD patients compared to HC, but the expression of miR-155-5p was modified by levodopa treatment, since a down-regulation of miR-155-5p in PD patients with the highest dosage was observed [18]. MiR-29a is involved in various neurodegenerative diseases, including AD and PD. Bai et al. reported that serum miR-29a was reduced in 80 PD patients compared with 80 HC and serum levels were higher in female patients and HC than male patients and HC [21]. Moreover, an upregulation of this miR was found in levodopa-treated PD patients compared to drug-naïve PD patients and healthy controls [22]. Finally, we also selected miR-106a-5p, that has been recently predicted to play a role in the pathogenesis of AD [23].

In our study, we found for the first time a significant increase of serum miR-34a-5p in PD male patients compared to PD female patients. miR-34a-5p was upregulated in plasmatic pure extracellular vesicles of 15 PD patients compared with healthy controls (HC) and its levels correlated with disease duration, Hoehn and Yahr and Beck Depression Inventory scores. Measuring miR-34a-5p levels in serum and not in extracellular vesicles in a larger sample of PD patients, we did not find correlations with clinical features. Differences in sample size, disease duration, antiparkinsonian treatment, in addition to the different methodological approach, may account for this discrepancy. [16]. Recently, Stefanik et al. found that hippocampal expression of miR-34a-5p was sex-dependent [24]. MiR-34a-5p is abundantly expressed in the brain and emerging evidence support its involvement in different neurodegenerative diseases as Alzheimer's disease and PD [13]. Moreover, Findeiss et al. observed that twelve miRNAS including miR-34a-5p were upregulated in α-synuclein-overexpressing Lund human mesencephalic neurons, a well-established cell model of PD, suggesting possible novel therapeutic targets for PD [25].

Neurodegeneration in PD results from a complex interplay of multiple immunological, inflammatory and genetic factors [26]. Certain genetic defects may contribute to microglial cell activation and production of inflammatory cytokines and chemokines, which finally lead to neurodegeneration [26]. Although dysregulation of miRNAs is only one of the disease-causing mechanisms that contribute to neurodegenerative disorders, evidence indicates that dysregulated miRNAs in NDs affect the severity and progression of neurodegenerative diseases [27]. MiRNAs not only affect gene expression inside the cells but also, when sorted into exosomes, systemically mediate the communication between different types of cells. Using miRTarBase, we predicted 85 target genes of miR-34a-5p and hypothesize that an increase of this miRNA can lead to the decreased expression of such predicted target genes. We used STRING to determine protein interactions among those genes involved in key biological processes such as regulation of neurogenesis, neuronal death and aging. Using open target platform, we identified 21 predicted target genes associated to PD. Among these target genes, AKT1, L1CAM and ATG5 may be particularly relevant. AKT1 is a serine/threonine-protein kinase responsible of the regulation of glucose uptake by mediating insulin-induced translocation of the SLC2A4/GLUT4 glucose transporter to the cell surface [28]. Sekar et al. observed a significant decrease of AKT1 in substantia nigra samples obtained from PD patients compared to the age-matched controls [28]. L1CAM is a neural cell adhesion molecule involved in multiple processes, including neuronal migration, axonal growth and synaptogenesis. In the mature brain, it plays a role in the dynamics of neuronal structure and function, including synaptic plasticity [29]. Recently, Cheng et al. found that L1CAM was downregulated in PD patients compared with HC. In addition, using LASSO model they observed that L1CAM was an immune hub gene for PD of their four-gene combined model [30]. Zhang et al. found that ATG5, a protein specifically required for autophagy, was downregulated in MPTP-induced PD mice model [31]. In addition, a significant downregulation of ATG5 in peripheral blood mononuclear cells (PBMCs) of PD patients compared with HC has been consistently reported [32, 33]. Moreover, Youn et al. reported significantly lower levels of the ATG5 protein in the cerebrospinal fluid samples of PD patients compared to HC [34].

In the present study, we also observed a significant up-regulation of miR-146a-5p in PD men compared with PD women. In addition, we observed a significant correlation between disease duration and miR-146a-5p specifically in male PD patients. The positive correlation between miR-146a-5p and disease duration may suggest a possible role of miR-146a-5p in the progression of disease in male patients. Using miRTarBase, we identified 47 target genes of miR-146a-5p and with STRING we determined protein interactions among those genes involved in neurogenesis, axon guidance and regulation of neurogenesis. Using an open target platform, we identified 17 predicted target genes associated with PD. Among these target genes, TGFB1, TLR2 and TLR4 may be of particular interest. Booth et al. found that TGFB1 was downregulated in iPSC-derived midbrain-patterned astrocytes from PD patients carrying the common *LRRK2* G2019S missense mutation [35]. Several studies reported an upregulation of TLR2 and TLR4 in PD patients, but no studies are available regarding gender differences [36, 37]. Normal and aggregated a-syn have shown TLR2- or TLR4-mediated microglial cells activation and neuronal loss in PD and mouse models [38, 39]. As for TLR4, regulation of hippocampal neurogenesis is region-specific in adult male mice while broader changes in neurogenesis throughout the hippocampus are found in female mice [40].

As a possible limitation of the study, we recognize that we have included only PD patients at a very early disease stage and did not include more advanced PD patients. Indeed, in this study we enrolled a cohort of levodopa-naive PD patients, thus explaining the short disease duration and low H&Y scores. The same cohort of patients is being followed up to evaluate gender differences in serum miRNAs after levodopa start.

In conclusion, our study supports the hypothesis that there are gender-specific differences in PD for miR-34a-5p and miR-146a-5p. A follow-up study of this cohort is underway to establish the possible role of these biomarkers in predicting disease progression and response to anti-parkinsonian treatments according to gender.

## Conflict of interest

The Authors declare no competing financial or non-financial interests directly or indirectly related to the work submitted for publication.

## Supplementary Information

Below is the link to the electronic supplementary material.

Supplementary file1 (ODS 8 KB)

## Data Availability

Data are available as supplementary information.
