# Correction to: Gender differences in microRNA expression in levodopa‑naive PD patients

**DOI:** 10.1007/s00415-023-11750-x

**Published:** 2023-05-08

**Authors:** A. Vallelunga, T. Iannitti, G. Somma, M. C. Russillo, M. Picillo, R. De Micco, L. Vacca, R. Cilia, C. E. Cicero, R. Zangaglia, G. Lazzeri, S. Galantucci, F. G. Radicati, A. De Rosa, M. Amboni, C. Scaglione, A. Tessitore, F. Stocchi, R. Eleopra, A. Nicoletti, C. Pacchetti, A. Di Fonzo, M. A. Volontè, P. Barone, M. T. Pellecchia

**Affiliations:** 1 Department of Life Sciences and Biotechnologies, Section of Medicines and Health Products, University of Ferrara, Ferrara, Italy; 2 Department of Medical Sciences, Section of Experimental Medicine, University of Ferrara, Ferrara, Italy; 3 Department of Medicine Surgery and Dentistry “Scuola Medica Salernitana”, Neuroscience Section, University of Salerno, Fisciano, Italy; 4 Department of Advanced Medical and Surgical Sciences, University of Campania “Luigi Vanvitelli”, Naples, Italy; 5 IRCCS San Raffaele, Rome, Italy; 6 Department of Clinical Neurosciences, Parkinson and Movement Disorders Unit, Fondazione IRCCS Istituto Neurologico Carlo Besta, Milan, Italy; 7 Neurologic Unit, AOU “Policlinico-San Marco”, Department of Medical, Surgical Sciences and Advanced Technologies, GF Ingrassia, University of Catania, Catania, Italy; 8 Parkinson’s Disease and Movement Disorders Unit, IRCCS Mondino Foundation, Pavia, Italy; 9 IRCCS Ca’ Granda Ospedale Maggiore Policlinico, Neurology Unit, Milan, Italy; 10 IRCCS San Raffaele Scientific Institute, Neurology Unit, Milan, Italy; 11 Department of Neurosciences and Reproductive and Odontostomatological Sciences, Federico II University, Naples, Italy; 12 IRCCS Istituto Delle Scienze Neurologiche di Bologna, Bologna, Italy; 13 University San Raffaele, Roma, Italy

**Correction to: Journal of Neurology** 10.1007/s00415-023-11707-0

The original version of this article unfortunately contained a mistake. First two authors should have been denoted as equally contributed to the paper.

